# A Plant-Produced Recombinant Fusion Protein-Based Newcastle Disease Subunit Vaccine and Rapid Differential Diagnosis Platform

**DOI:** 10.3390/vaccines8010122

**Published:** 2020-03-09

**Authors:** Fanshu Ma, Erqin Zhang, Qingmei Li, Qianru Xu, Jiquan Ou, Heng Yin, Kunpeng Li, Li Wang, Xiangyue Zhao, Xiangxiang Niu, Xueyang Li, Shenli Zhang, Yanan Wang, Ruiguang Deng, Enmin Zhou, Gaiping Zhang

**Affiliations:** 1 Department of Preventive Veterinary Medicine, College of Veterinary Medicine, Northwest A&F University, Yangling 712100, China; marfanshu@nwafu.edu.cn (F.M.); xuqianru@nwafu.edu.cn (Q.X.); zhouem@nwsuaf.edu.cn (E.Z.); 2 Department of Preventive Veterinary Medicine, College of Animal Science and Veterinary Medicine, Henan Agricultural University, Zhengzhou 450002, China; zhangerqin76@163.com (E.Z.); nxx188@foxmail.com (X.N.); lxy4241@foxmail.com (X.L.); zhangshenli203814@foxmail.com (S.Z.); 3 Key Laboratory of Animal Immunology, Henan Academy of Agricultural Sciences, Zhengzhou 450002, China; liqingmeis@gmail.com (Q.L.); nongkewangli@163.com (L.W.); xiangyuezhao@foxmail.com (X.Z.); yanan18@mails.jlu.edu.cn (Y.W.); rgd999@163.com (R.D.); 4 Wuhan Healthgen Biotechnology Corp., Wuhan 430074, China; jqou@oryzogen.com (J.O.); yhen@oryzogen.com (H.Y.); lkp@oryzogen.com (K.L.)

**Keywords:** Newcastle disease virus, fusion protein, rice endosperm, vaccine, plant-produced, DIVA, immunochromatographic strip

## Abstract

Newcastle disease (ND) is a highly contagious avian disease, causing considerable economic losses to the poultry industry. To obtain a safe, inexpensive, and effective ND vaccine to meet the international trade requirements of differentiating infected from vaccinated animals (DIVA), here we report the production of Oryza sativa recombinant fusion (F) protein in stably transformed transgenic rice seeds via agroinfiltration. The F protein expression level was enhanced 3.6-fold with a genetic background in low glutelin. Inoculation of plant-produced F antigen into Specific Pathogen Free (SPF) chickens markedly elicited neutralizing antibody responses against homologous and heterologous ND virus strains. Two doses of 4.5 μg fully protected chickens from a lethal ND challenge without any clinical symptoms. The mean weight gain of F protein-immunized chickens within 15 days after challenge was significantly higher than that of traditional whole virus vaccine-immunized chickens, thereby obtaining higher economic benefits. Moreover, the sera from the chickens vaccinated with the plant-produced F vaccine did not show reactivity in an immunochromatographic strip targeting the haemagglutinin-neuraminidase protein (HN) protein, and DIVA could be achieved within 10 minutes. Our results demonstrate that the plant-derived F vaccine along with immunochromatographic strips could be useful in the implementation of an NDV eradication program.

## 1. Introduction

Newcastle disease (ND), an acute and highly contagious avian disease caused by avian paramyxovirus, remains endemic throughout Africa, Asia, and Europe, with frequent epidemics occurring despite the availability of global commercial vaccinations since the 1930s [1,2]. When the virus is introduced into a susceptible flock, including domestic poultry, virtually all the birds will be infected within two to six days (World Organisation for Animal Health, OIE, 2019). According to the World Animal Health Information Database (WAHIS), an average of 60 countries reported NDV outbreaks yearly from 2013 to 2018, and the number of viral genotypes is increasing (OIE, 2019), which might be related to the mutation and reconstitution of live vaccines [3]. Therefore, ND continues to be a threat and to cause considerable economic losses to the poultry industry worldwide. ND in its highly pathogenic form is listed in the World Organization for Animal Health (OIE) Terrestrial Animal Health Code and must be reported to the OIE.

Due to the constant threat of introduction of the virus from wild birds, vaccination and the establishment of poultry biosecurity are essential to the prevention of ND. At present, ND vaccines are available in live, inactivated forms and are licensed for use in many countries worldwide. Where vaccination is carried out, serological surveillance is of limited value because it cannot discriminate between NDV infection and vaccination. Positive NDV antibody test results can have four possible causes: (i) natural infection with NDV; (ii) vaccination against ND; (iii) exposure to vaccine virus; and (iv) maternal antibodies. This is a challenge for ND eradication plans in many countries. Chicken-producing countries that are vaccinated with whole virus are adversely affected by restrictions on international trade designed to avoid the importation of chickens that may be infected with NDV into ND-free zones [4]. Therefore, the development of the next generation of NDV vaccines is essential to distinguish between infected and vaccinated animals while ensuring disease control. As an alternative to live and inactivated vaccines, subunit vaccines require only an immunogenic portion of the target virus, without the risk of virus spread and recombination, and can be designed to develop a differentiating infected from vaccinated animals (DIVA) strategy and disease eradication plan for ND control. The greatest dilemma in avian vaccine research is the balance between product prices and immunogenicity. Currently, the prices of NDV inactivated vaccines and live vaccines used in China are much lower than 0.1 RMB per dose. Traditional expression systems such as insect cells are not easily accepted by the market, and low-cost *E. coli* expression systems cannot readily obtain highly immunogenic antigens; thus, there are currently no NDV subunit vaccines on the market.

The envelope of NDV contains two transmembrane glycoproteins, the haemagglutinin-neuraminidase protein (HN) and the fusion protein (F), which form spike-like protrusions on the outer surface of the virion. The HN protein is responsible for the attachment of the virus to the sialic acid receptor on the host cell and for releasing progeny virions from the surface of infected cells [5,6]. Fusion protein (F) glycoprotein is an important protective protein of NDV that promotes the merger of viral and cellular bilayers and the opening of a pore to deliver the viral genome into the cytoplasm of the host [7]. These proteins are the two major vaccine antigen candidates. F glycoprotein has been shown to be the major contributor to the induction of neutralizing antibodies and protective immunity, followed by the HN protein, which conferred partial protection against an intravenous challenge [8,9]. Therefore, compared with the HN protein, the F protein is more ideal as a subunit vaccine antigen.

The “transgenic plant vaccine” was proposed in 1992 [10], and transgenic plants are promising vehicles for recombinant proteins [11,12,13]. Compared to traditional systems, plant hosts exhibit easy scalable production, very low production costs, high production quality, lack of pollution, and the process of eukaryotic protein modification [14,15]. Moreover, vaccines produced by plants avoid the culture of viruses and bacteria, eliminating the risk of infection [16]. A number of pharmaceutical proteins produced by transgenic plants are currently in clinical development. ZMapp antibodies against the Ebola virus and influenza vaccines have shown the great potential of the plant system.

Rice seeds are a cost-effective bioreactor for the large-scale production of pharmaceuticals [14]. To obtain inexpensive and effective subunit ND vaccines to meet market needs, in this study, we expressed an *Oryza sativa* recombinant F protein from transgenic rice seeds. The expression level of the F protein was increased by hybridizing F-transgenic rice with low-gluten rice. Immunoassays and protective assays have demonstrated that recombinant proteins do not trigger a stress response, and 4.5 μg immunization doses protect chickens from lethal viruses. This was the first time that the immunogenicity of the plant-made F vaccine was comprehensively characterized in vivo. Moreover, plant-produced F vaccine enables the differential diagnosis of vaccination and natural infection by detecting HN-specific antibodies. Our results demonstrate that transgenic rice engineering is a promising approach for the future production of an affordable ND vaccine.

## 2. Materials and Methods 

### 2.1. Construction of Plant Vector and Rice Genetic Transformation

The DNA sequence coding for the F gene (GenBank accession No. JN618348.1) was synthesized by GenScript Corporation using rice codon preferences. The F gene was subcloned into the *MlyⅠ-Xho* Ⅰ site of intermediate vector pMP3 containing the Gt13a promoter, a signal peptide and the *t-nos* terminator (Healthgen Biotechnology Co., Ltd., Wuhan, China). The recombinant plasmid pMP3-F was digested by *Eco*R Ⅰ and *Hind* Ⅲ and cloned into the plant vector pCAMBIA1300 (Healthgen Biotechnology Co., Ltd., Wuhan, China), which includes the hpt Ⅱ (hygromycin resistance) gene as a selective marker and the right and left borders necessary for T-DNA transmission. The plasmid pCAMBIA1300-F was transformed into the callus regenerated from rice cultivar TP309 by *Agrobacterium*-mediated transformation as described previously [14]. Positive callus was obtained after hygromycin-resistance screening and transplanted into the greenhouse.

### 2.2. PCR Analysis to Select Positive Transgenic Plants

Total genomic DNA was extracted from young leaves of transgenic rice by the CTAB method. Positive transgenic plants were doubly identified by the hygromycin gene and the F gene. The F gene was amplified with forward primer (5’-CACATCCATCATTATCCATCCACC-3’) and reverse primer (5’-GAGGAGGGTGGTGAGGGT-3’). The *hyg* gene was amplified with forward primer (5’-CGATTCCGGAAGTGCTTGAC-3’) and reverse primer (5’-CGTCTGCTGCTCCATACAAG-3’).

### 2.3. SDS-PAGE and Western Blotting

The transgenic rice extract and the pure F protein were separated on a 12% polyacrylamide gel and then transferred to a PVDF membrane (Millipore, Darmstadt, Germany). The membrane was blocked with 5% skim milk in phosphate-buffered saline Tween 20 (PBST) buffer to prevent non-specific reactions. After 2 h, the Western blot membrane was washed 3 times with PBST buffer and incubated with a monoclonal antibody 13A5 to the F protein [17] at a dilution of 1:1000. Subsequently, a goat anti-mouse antibody (1:8000) conjugated with HRP (Abbkine, Beijing, China) was reacted with a monoclonal antibody. Finally, the hybridization reaction was detected by chemiluminescence detection.

### 2.4. F Protein Purification

The rice seeds were ground into a powder and extracted with phosphate buffer (25 mM PB with 50 mM NaCl, pH 6.3) in a 1:6 (wt/vol) ratio at room temperature for 2 h with constant stirring. The seed residue and precipitate were removed by centrifugation at 10,000 rpm for 30 min at 4 ℃. The supernatant was aspirated, and the conductance value and pH were measured. The pH of the extract supernatant was adjusted to 5.0 with 1 M hydrochloric acid to precipitate some of the impurities. The sediment was discarded by filtration with a 0.8 to 0.22 μm filter. Then, the clarified extract was loaded into an Capto^TM^ MMC purification column (GE Healthcare, Boston, MA, USA) with 25 mM PB buffer (25 mM PB with 50 mM NaCl, pH 5.0) and eluted with 1 M sodium phosphate dibasic (pH 9.0). The resulting collected fractions were purified with Q Bestarose Fast Flow (Bestchrom, Shanghai, China). At this time, the F protein flowed out of the Q column to be separated from the impurities bound to the column. The F fractions from Q Bestarose were finally purified through a Superdex 75 pg column (GE Healthcare, Boston, MA, USA). The purity of F was determined by SDS/PAGE.

### 2.5. Enzyme-Linked Immunosorbent Assay (ELISA)

The F protein concentration in the extract was measured by ELISA quantitative assay. Briefly, chicken anti-NDV IgG was diluted 1:500 in carbonate buffer (pH 9.6) and then applied to a 96-well microwell overnight at 4 °C. After washing 3 times with PBST, the plate was blocked with 5% skim milk at 37 °C for 2 h. The rice extract and the pure F protein standard were each subjected to gradient dilution and incubated at 37 °C for 1 h. The nontransgenic plant TP309 was set as a negative control. After washing 5 times, an anti-F protein mouse monoclonal antibody 13A5 was added to the well and incubated for 1 h at a dilution of 1:1000. Subsequently, a goat anti-mouse IgG/HRP antibody (Abbkine, Beijing, China) was added, and the reaction was carried out at 37 °C for 1 h. After washing five times, tetramethylbenzidine (TMB) substrate (Sigma-Aldrich, Darmstadt, Germany) was added to each well. After 10 min, the OD_450_ value was read with a microplate reader. A standard curve was drawn based on the OD value and the concentration of the standard F protein, and then the total soluble protein (TSP) content was determined from the OD value of the sample.

### 2.6. Animal and Vaccine

All chickens in the experiment were four-week-old SPF chickens (n = 80) purchased from Beijing Boehringer Biotechnology Co., Ltd., (Beijing, China) The chickens were acclimated for one week prior to the beginning of the vaccine study. The chickens were numbered individually and randomly assigned in isolators into nine treatment groups. To verify that the four-week-old SPF chickens had no prior exposure to NDV, twenty chickens randomly selected prior to the start of the trial were bled, and the sera were tested using the Newcastle disease virus Antibody Test Kit (BioChek, Reeuwijk, The Netherlands) according to the manufacturer’s instructions. The samples to positive control ratio (S/P) values (0.08 to 0.21) were negative (S/P < 0.3) in all cases.

The plant-derived F vaccine was prepared by mixing the purified F protein and an adjuvant. The purity of the F protein after three purification steps reached 95%. According to the vaccine dose in each group, the pure F protein produced by plants was diluted in PBS and mixed with Montanide™ ISA 71 VG adjuvant (Seppic, Paris, France) at a ratio of 3:7. The commercial live vaccine LaSota was purchased from the manufacturer. According to the label, the EID50 of the commercial vaccine (HARVAC, Harbin, China) is ≥ 10^6^ per recommended dose (0.5 mL) for initial immunization or booster injections.

### 2.7. Efficacy Study in Chickens

In the nine experimental groups, six groups were intramuscularly (IM) inoculated with different doses of plants to produce F protein (0.5 μg, 1.5 μg, 4.5 μg, 9 μg, 18 μg, and 36 μg) of 100 µL each. The seventh and eighth groups were intramuscularly inoculated with the same volume of non-transgenic rice TP309 and PBS as two negative controls. The ninth group was vaccinated with 50 µL of commercial live vaccine (LaSota strain) by eye drop as a positive control. At day 0 before the vaccination, 1 mL of blood was sampled from the wing vein of each of ten randomly selected chickens to confirm that the SPF chickens had no prior exposure to NDV. All treatment groups were boosted with the respective vaccine 28 days after the initial vaccination. Sera were collected on days 7, 14, 21, and 28 post inoculation after the first and booster immunizations to determine the level of antibody response to treatment. Chickens were routinely monitored daily for the first 7 days after immunization to assess safety. Lesions at the injection site, animal behavior, and body weight were also recorded. F-specific antibodies were detected with a commercial Newcastle Disease Virus Antibody Test Kit (BioChek, Reeuwijk, The Netherlands) according to the manufacturer’s instructions. The sample to positive control ratio (S/P) was calculated from the optical density at OD_450_ for each sample. S/P > 0.3 is considered positive. The virus challenge experiment was performed 28 days after booster immunization. The chickens were challenged via the oculo-nasal route with a 50% egg infectious dose (EID_50_) of 10^6.5^ of the highly virulent NDV gene type Ⅶ strain XX-08. Chickens were observed daily throughout the trial for clinical signs of disease until 15 days post challenge.

### 2.8. Virus Neutralization Assay

To verify the ability of the vaccine to resist NDV viruses of different strains, we selected an F48E8 strain homologous to the plant-produced vaccine and an XX-08 strain heterologous to the vaccine produced by the plant for neutralization experiments. First, BHK21 cells showing good growth were plated into 96-well cell plates and incubated at 37 °C in a CO_2_ incubator for 16 h. Subsequently, 50 μL of 100 TCID_50_ NDV F48E8 strain and XX-08 strain were mixed with an equal volume of 2-fold serially diluted chicken sera in a 96-well plate. Both virus and serum were diluted in serum-free DMEM. After incubation at 37 °C for 1 h, virus and serum mixtures were added to 96-well cell plates in sequence. The 96-well plate was incubated for 2 h to allow the virus to fully infect the cells, and then the solution in the well was discarded, and the cell plate was gently washed twice with sterile PBS. Then, 100 μL of Dulbecco’s modified Eagle Medium (DMEM) containing 2% fetal bovine serum (FBS) was added to each well and cultured at 37 °C in a CO_2_ incubator. The cell plate was removed at 48 h. The cells were fixed by adding 100 μL of pre-cooled absolute ethanol to each well. Immunocytochemical staining of NDV in cells was performed as with anti-HN protein monoclonal antibody 5F2 and goat anti-mouse IgG/HRP antibody (Abbkine, Beijing, China). The virus staining results were observed under a microscope. When the antibodies in the serum completely neutralize the virus, there is no cell staining in the wells. Conversely, when neutralizing antibodies do not completely neutralize the virus, red-stained cells appear in the wells. The neutralizing antibody titer in the serum sample is expressed as the reciprocal of the highest dilution that causes 100% neutralization. 

### 2.9. Immunochromatographic Strip

We previously developed two immunochromatographic strips, which are used to detect antibodies that recognize HN protein and F protein of NDV, respectively. The recombinant HN or F proteins were coated in an antigen pad. The anti-HN mAb or anti-F mAb was labeled with colloidal gold as the detector. A chicken anti-NDV polyclonal antibody and staphylococcal protein A (SPA) were blotted on the nitrocellulose membrane for the test and control lines, respectively. Chicken serum under different immunization conditions was added into the sample well of the strip. In the absence of a specific antibody in the serum, when the serum flowed through the antigen pad, the recombinant antigen was released and combined with the AuNPs-labeled mAb to form a complex. The complex was then captured by the polyclonal antibody immobilized on the test-zone and appeared as a red band. If specific antibody existed in the serum, the antibody in the serum would compete against the captured antibody on the test zone to specifically bind to the limited recombinant antigen. The test zone did not appear as a red bond. The results of the test-line (T) and the control-line (C) were recorded. Two red lines indicate a negative result, and only the C-line indicates a positive result. 

### 2.10. Ethics Statement

All animal experiments involved in this study were carried out under the approval of the Animal Experimental Committee of Henan Academy of Agricultural Sciences, with ethic approval number LLSC4102019058. According to Chinese animal ethics procedures and guidelines, all animals received humane care.

## 3. Results

### 3.1. Generation and Identification of NDV-F Transgenic Lines in Rice Seeds

**Figure 1:**
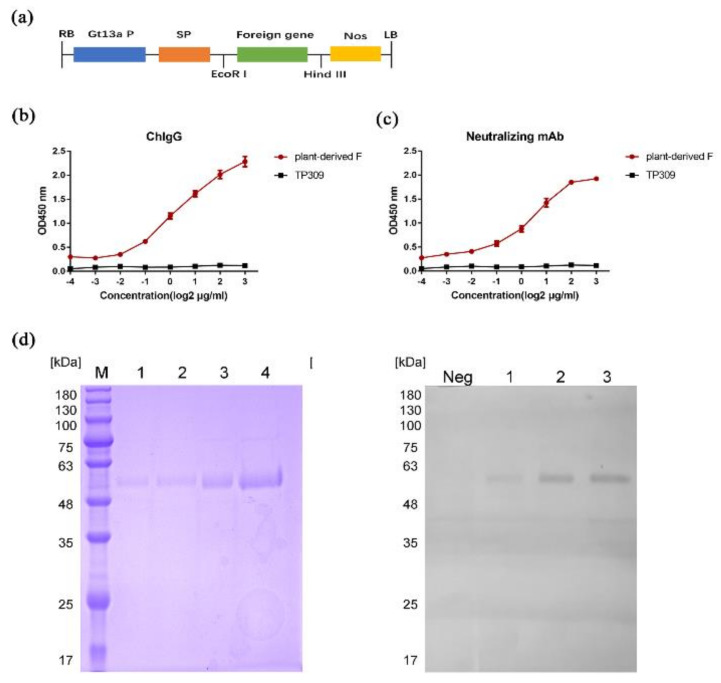
The production and purification of plant-made fusion (F) protein. (**a**) Schematic representation of the pCAMBIA 1300-F plasmid for the generation of transgenic rice lines. RB, right border; Gt13a promoter, rice seed storage protein glutelin gene promoter; SP, Gt13a signal peptide; Nos, nopaline synthase gene terminator; LB, left border. (**b**) Binding of chicken Newcastle disease (ND)-positive IgG to F protein was measured by ELISA (n = 3). (**c**) Binding of 13A5 mAb that recognizes the neutralizing epitope to F protein was measured by ELISA (n = 3). (**d**) SDS-PAGE electrophoresis and Western-blot of purified plant-produced F protein. Lanes 1–4 and F1–F2 were twofold serial dilutions of F protein. Neg was non-transgenic rice.

To obtain high-level expression of the recombinant HN-dimer in rice seed, the F gene sequence was synthesized using rice-preferred codons followed by the introduction of a strong endosperm-specific promoter Gtl3a and its signal peptide to target the F protein into the protein storage vacuoles (Figure 1a). Agrobacterium tumefaciens-mediated pCAMBIA1300-F was introduced into the rice callus, and 101 plants were found to be positive for the expression of F and hygromycin genes. A total of 64 independent transgenic plants were obtained using Agrobacterium-mediated transformation. To determine the expression of NDV-F in transgenic seeds, sodium dodecyl sulfate polyacrylamide gel electrophoresis (SDS-PAGE) and Western blot analysis revealed a predominant protein band of approximately 55 kDa in transgenic grains, which was not detected in the TP309 control rice seeds (Figure 1d). The plant-expressed F protein was recognized specifically by the neutralizing monoclonal antibody 13A5 and chicken NDV-positive IgG, indicating that the epitope of F protein expressed by plants is correctly displayed (Figure 1b,c).

**Figure 2:**
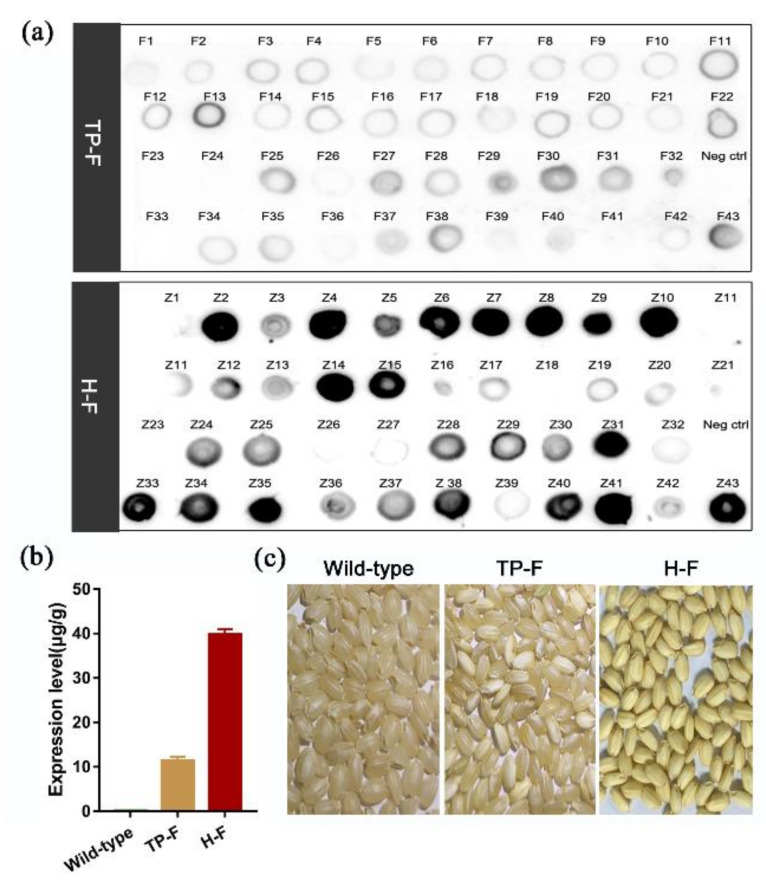
Comparison of hybrid rice expressing the F protein (H-F) and transgenic TP309 rice expressing the F protein (TP-F). (**a**) F protein was detected in TP309 transgenic lines (TP-F) and low-glutelin transgenic lines (H-F) by dot blot using the anti-F Abs. Each dot represents a different transgenic line. Neg ctrl indicates the negative control of non-transgenic rice TP309. (**b**) The expression levels of H-F and TP-F in rice seeds as quantified using ELISA. (**c**) Phenotype of F-expressing seeds in hybrid transgenic rice (H-F; right), transgenic rice of TP309 (TP-F; middle) and non-transgenic seeds (left).

To increase the expression of the F protein, we hybridized TP309 rice expressing the F protein (TP-F) with low-gluten rice to obtain a hybrid rice expressing the F protein (H-F). The expression of F protein in rice seed extract after hybridization was significantly increased 3.6-fold (Figure 2a,c). The seeds of the hybrid line H-F displayed an opaque phenotype compared with wild-type seeds and TP-F seeds (Figure 2b). We monitored the genetic stability of the transgenic line H-F-1 and found that the expression of F was stable from the T2-T3 generation. Thus, this transgenic line was used for further studies.

### 3.2. Purification of F Protein from Rice Seeds

To achieve correct folding of the antigen and satisfy the requirements of vaccine antigen design worldwide, plant-produced F protein is tag-free. The complete purification process takes ∼60 h. The processing comprises extraction, preliminary purification, and three chromatography steps with Capto-MMC, Q-Sepharose, and gel filtration, followed by concentration. The preliminary purification requires adjustment of the pH of the extraction from 6.0 to 5.0 to precipitate impurities for 3 h at Room temperature (RT). The color of the rice seed extract was changed from cloudy greyish white to clear pale yellow by stepwise filtration through different pore size filters. After three chromatographic purification steps, the F protein final product showed a single peak in a reverse-phase HPLC analysis, and other proteins were barely visible in SDS-PAGE (Figure 1d).

### 3.3. Plant-Produced F Induced Potent Immune Response in Chickens Without Affecting Weight Gain

**Figure 3:**
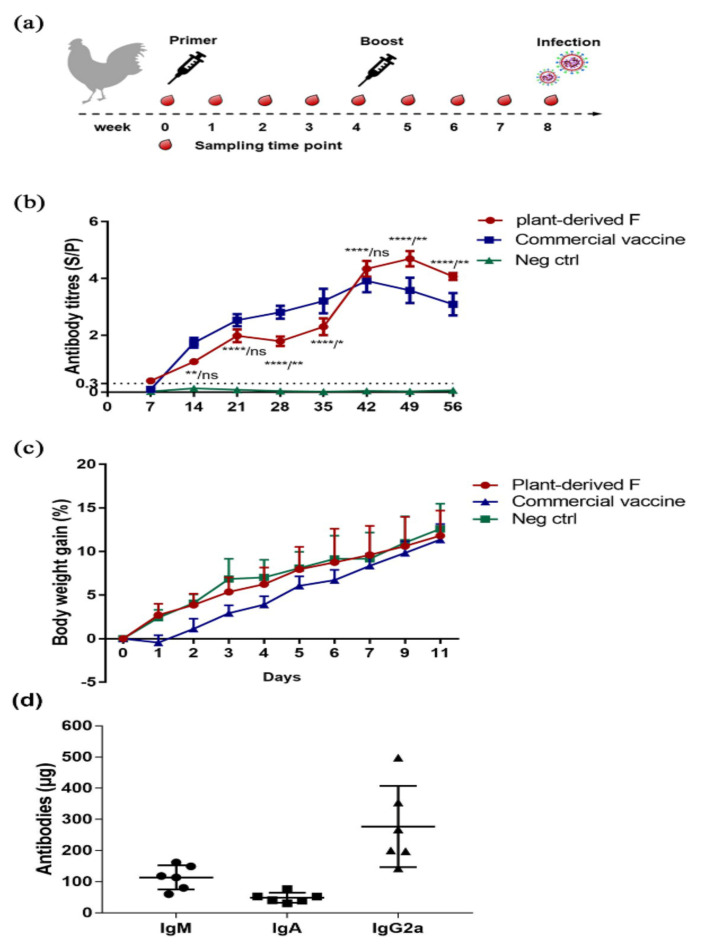
Humoral immune responses of plant-produced F. (**a**) Scheme of immunization, virus infection, and sampling. (**b**) The F-specific antibody titers in the plant-produced F vaccine and commercial live virus vaccine (LaSota strain) were detected by ELISA kits. According to the instructions, the cut-off value of S/P is 0.3. Data are shown as the means ± SEM from ten chickens. Statistical analysis was performed by two-way ANOVA. Differential markers are labelled according to the significant difference between plant-produced F protein and non-transgenic rice/significant difference between plant-produced F protein and commercial live virus vaccine, where “ns” represents no significant difference, * indicates *p* < 0.05, ** *p* < 0.01, and *** *p* < 0.001. (**c**) Weight gain after vaccination with plant-derived F, PBS and commercial live vaccine. (**d**) Analysis of antibody subtypes in sera from the chicken vaccinated with the plant-produced F vaccine. IgM are shown as circles, IgA as squares, and IgG2a as triangles.

To determine the immunogenicity of F protein expressed in rice, thirty 4-week-old SPF chickens were randomly divided into three groups. Ten chickens in treatment group A were inoculated two times at 4-week intervals with 18 μg of plant-derived F protein adjuvanted with Montanide^TM^ ISA 71 VG (Seppic, Paris, France), and group B was vaccinated with live virus vaccine of the LaSota strain as the positive control. Group C was vaccinated with non-transgenic rice TP309. All three groups were vaccinated two times at an interval of 4 weeks (Figure 3a). The F protein-specific antibodies were detected by commercial ELISA kits (Figure 3b). Within 21 days after the first injection, the antibody response produced by the commercial live vaccine was comparable to that of the rice-derived vaccine, and there was no significant difference. The plant-derived F vaccine induced a potent antibody response through the boost injection. The antibody titer reached a maximum of 4.67 (S/P > 0.3 was positive) in the third week after the second immunization, while that of the commercial vaccine was 3.581. Interestingly, the antibody titer of the plant-derived F group was significantly higher than the commercial live vaccine at the third and fourth weeks after the second immunization (*p* < 0.01). Antibody isotype analysis showed the antibodies were biased toward IgG2a at 28 days after immunization. In addition, IgM and IgA were also present in the antibody (Figure 3d).

To determine the safety of rice-produced F protein administered intramuscularly (IM), we evaluated weight gain after vaccination with plant-derived F and commercial live vaccine of the LaSota strain. Chickens immunized with plant-produced F vaccine did not show adverse clinical reactions. The weight of chickens after inoculation were similar to those of PBS-immunized groups, but the live virus vaccine group gained weight slowly in the seven days after vaccination (Figure 3b) and then returned to normal.

### 3.4. The Optimization of Vaccine Immunization Dose

**Figure 4:**
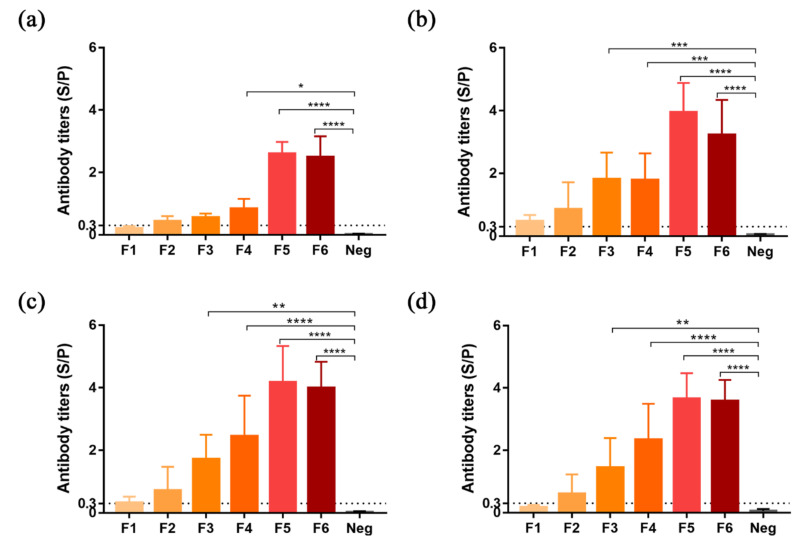
Immune response in chickens after receiving booster vaccination at different antigen doses. Antibody titers of different antigen dose groups on the 7th (**a**), 14th (**b**), 21st (**c**), and 28th (**d**) days after booster immunization. The F1 to F6 groups were inoculated with 0.5 μg, 1.5 μg, 4.5 μg, 9 μg, 18 μg, and 36 μg, respectively. The negative group (Neg) was inoculated with 36 μg of non-transgenic rice extract. *p* < 0.05 (*), *p* < 0.01 (**), *p* < 0.001(***), *p* < 0.0001 (****).

To determine the relationship between antibody titer and vaccination dose, we divided sixty 4-week-old SPF chickens into six groups (F1 to F6) and inoculated them with 0.5 μg, 1.5 μg, 4.5 μg, 9 μg, 18 μg, and 36 μg of plant-derived F protein in sequence. According to the F-specific IgG, from 1 week to 4 weeks after booster immunization, the antibody titer was positively correlated with the immunization dose between 0 and 18 μg. Above 18 μg, the antibody titer decreased as the antigen dose increased further. Among the six groups of different doses of F protein, one and four weeks after booster immunization (Figure 4a, 4d), the ELISA results of the F1 group (0.5 μg/dose) were negative, while the antibody titers at two and three weeks were slightly higher than the threshold (S/P values of 0.47 and 0.31 > 0.3) (Figure 4b, 4c). The antibody titer of the F2 group was higher than the threshold within 4 weeks (S/P values were 0.44, 0.85, 0.71, and 0.61) but was not significantly different from that of the non-transgenic rice group (from 0.02 to 0.05). However, the antibody titers of F3 to F6 (4.5 μg to 36 μg) were significantly higher than those of the non-transgenic rice group. Among them, the antibody titer of the F5 group was the highest, and the S/P values detected at the four time points were 2.60, 3.95, 4.17, and 3.65, respectively.

### 3.5. Serum Neutralizing Antibodies against Homologous and Heterologous ND Strains

**Figure 5:**
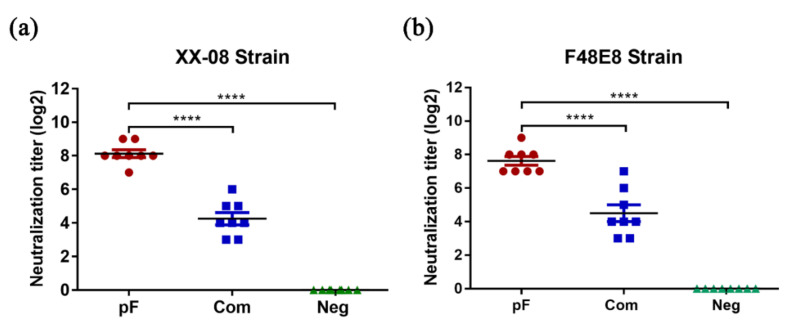
Neutralizing antibody titers in chicken sera before virus challenge were detected by two genotypes of NDV. The XX-08 strain is homologous (**a**) and F48E8 (**b**) is heterologous to the plant-produced F. The pF group (red) was vaccinated with plant-produced F protein. The Com group (blue) was inoculated with commercial live vaccine (LaSota strain). The neg group (green) was inoculated with non-transgenic rice. *p* < 0.0001 (****).

Since a crucial factor in the prevention of virus infection is the production of neutralizing antibodies that block viral infection to target cells, we evaluated neutralizing antibodies to two different NDV strains in treated animals at the same time to evaluate the efficacy of rice-produced vaccine against NDV. Chicken immune sera were collected before the virus challenge (the 28th day after booster immunization). The diluted sera were incubated with homologous NDV strain (XX-08) and heterologous NDV strain (F48E8) separately to detect the neutralizing antibodies in the sera by microwell neutralization experiments. The neutralization antibody titers produced by the plant vaccine to the homologous virus ranged from 7log2 to 9log2 (geometric mean titer (GMT) of 8.125 log_2_) (Figure 5a) and to the heterologous virus with the GMT of 7.6log2 (Figure 5b), which were significantly higher than those of the commercial live virus vaccine (to xx-08 strain with 4.25log2, and F48E8 strain with 4.5log2). 

### 3.6. Plant-Produced F Protected Chicken from NDV Challenge

**Figure 6:**
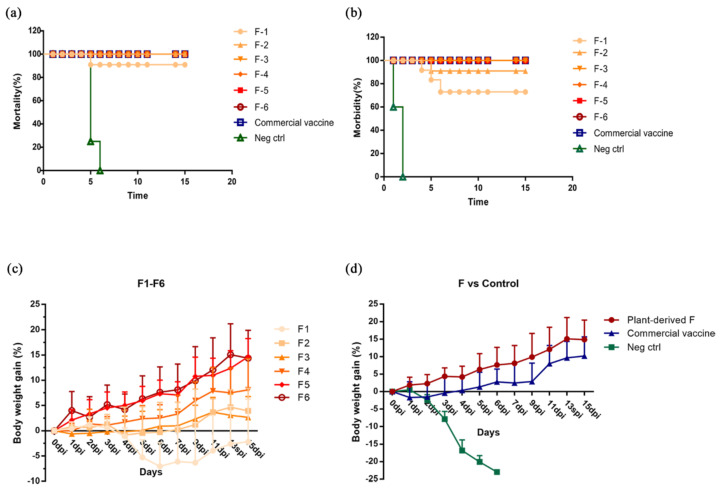
Protective efficacy against infection by NDV. Immunized chickens were challenged with a lethal dose (10^6^ EID50) of the XX-08 strain. Body weight, survival rate, and morbidity changes were monitored daily for 15 days. (**a**) Survival rate and (**b**) clinical conditions of vaccinated chickens in all groups. (**c**) Weight gain over 15 days post challenge in chickens administered plant-produced F vaccines at different antigen doses. (**d**) Weight gain over 15 days post challenge in F6 and commercial live vaccine groups (LaSota strain).

We examined the ability of the plant-derived NDV-F vaccine to confer protection following NDV exposure. Chickens were inoculated with six doses from 0.5 to 36 μg and challenged 28 days post vaccination with a lethal dose (10^6.0^ EID_50_) of the XX-08 strain by eye-dropping. Body weight and clinical presentation were monitored up to once daily for the duration of the study. Chickens in the non-transgenic group began to exhibit depression, swollen eyelids, opaque eyes, and loss of appetite on the second day post inoculation (dpi); symptoms of asthma and salivation occurred at 3 dpi. Six of the 10 chickens died at 4 dpi, and all chickens in the non-transgenic rice group died at 5 dpi (Figure 6a). In six different doses of plant-derived F groups, the vaccine protection rate gradually increased from 72.9% to 100% with increasing immunization dose. In the six groups, Group 1 (0.5 μg) and Group 2 (1.5 μg) did not provide full protection against the challenge. In Group 1, one chicken died on the fifth day after infection (5 dpi), while two chickens developed diarrhea at 4 dpi and 5 dpi. In Group 2 (1.5 μg), one chicken developed diarrhea and depressive symptoms in the first three days and then returned to normal, but none of the chickens died (Figure 6a, 6b). Chickens in Group 3 (4.5 μg) to Group 6 (36 μg) were completely protected against challenge without any clinical symptoms. In addition, the weight gain of F protein-immunized chickens was 14.8% within 15 days after the challenge, which was higher than that obtained for traditional whole virus vaccines (10.1%) (Figure 6d). Taken together, these data provide evidence that both plant and commercial live vaccines protected chickens from ND clinical signs and prevented clinical presentation and death following wild-type NDV challenge.

### 3.7. Distinction Between Infected and Vaccinated Animals

**Figure 7:**
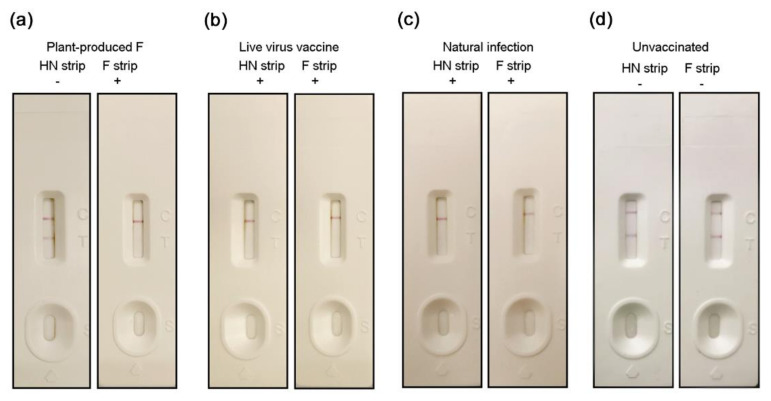
Distinction between infected and vaccinated animals. The specific antibodies that recognize HN and F proteins were detected by immunochromatographic strips. (**a**) Serum from chickens vaccinated with plant-produced F. (**b**) Serum from chickens vaccinated with ND live virus vaccine. (**c**) Serum from chickens infected with NDV. (**d**) Serum from unvaccinated and uninfected chickens.Two lines represent negative and one line represents positive.

To distinguish between naturally infected chickens and vaccine-immunized chickens, we have previously developed immunochromatographic strips that can rapidly detect antibodies that specifically recognizes the HN and F protein antibodies. For the sera of chickens inoculated with the plant-produced F, the result of HN antibody test strip was negative, while the F antibody strip was positive (Figure 7). For naturally infected chicken sera, both HN and F antibodies were positive. Commercial live vaccines have the same results as natural infections. Therefore, chickens vaccinated with whole virus cannot be distinguished from naturally infected chickens. 

## 4. Discussion

NDV is a major viral disease that severely restricts the development of the global poultry industry [18]. The control of ND must include strict biosecurity that prevents virulent NDV from contacting poultry, as well as the stringent and appropriate administration of vaccines. Currently, the effective containment of ND outbreaks is normally achieved with the utilization of a combination of vaccinations, rapid diagnostic assays, and culling of infected flocks. From the early 1950s, live and inactivated ND vaccines were the only vaccine platforms available for most developing countries and were used to decrease economic losses resulting from morbidity and mortality. Due to the severe production cost constraints of avian vaccines, genetically engineered vaccines such as subunit vaccines are currently only at the research stage and are not recognized by poultry farmers. Plant molecular farming offers a cost-effective and scalable approach to the expression of recombinant proteins, providing an alternative to conventional production platforms for developing countries [4]. Therefore, this study was conducted in a rice expression system to obtain low-cost and efficient recombinant antigens in rice endosperm.

The strains of ND can be divided into two classes. Viruses from class I mainly infect water-fowl and captured wild birds. Class II viruses are present in both wild birds and domestic poultry and are further divided into 16 genotypes based on the sequence and phylogenetic analysis of the F protein gene. Genotype VII of class II NDV is the main strain that has caused outbreaks in Europe, south America, the Middle East, and Africa in recent years [1,19]. Therefore, the F protein gene design in this study was based on genotype VII as the backbone [20]. By comparing the 32 strains of ND in GenBank, we replaced the highly mutant sequence of genotype VII to increase the resistance of the plant vaccine to heterologous ND. As the main storage protein of rice seeds, glutelin accounts for approximately 60% to 80% of total endosperm protein. Low-storage protein mutants provide more space for the accumulation of foreign gene products than the normal host plant [21], allowing higher accumulation of foreign gene products. To enhance the expression of the F protein, we hybridized TP309 rice expressing the F protein (TP-F) with low-glutelin rice to obtain the hybrid rice H-F. After hybridization, the expression of F protein can be increased 3.6-fold.

Recently, rice seeds have been demonstrated to be an effective bioreactor for molecular pharming [14,22]. The rice endosperm provides the ideal environment, as the seed not only provides a stable site for the deposition of recombinant proteins to prevent degradation by proteases but also can be stored at ambient temperatures for several years [23]. The N-glycosylation of α-1,3-fucose in seed cells was as low as 10%, and the N-glycosylation patterns of the proteins in endosperm cells were much simpler than those in leaf cells [22]. Lower α-1,3-fucose contents might make rice seed a much more favorable platform for the production of recombinant proteins. In this study, we compared the weight change and clinical symptoms of the live virus ND vaccine group and the plant-produced F vaccine group after immunization. Chickens immunized with plant-produced F vaccine did not show adverse clinical reactions. The weight of chickens after inoculation were similar to those of PBS-immunized groups, but the live virus vaccine group gained weight slowly in the seven days after vaccination (Figure 3b).

DIVA is important for international trade and disease control. The country is obliged to report to the OIE when the poultry is infected with virulent ND. Trading partners may suspend imports of poultry or poultry products from that country. Whole virus vaccines elicit immune responses against the full complement of viral proteins, making it difficult to distinguish between immunized animals and vaccinated animals by serological methods. Among them, HN and F, as the main protective antigens, can elicit a strong antibody response in chickens. Plant-produced F vaccine does not contain HN protein, and no genetic material exists. Therefore, vaccinated chickens can be distinguished by detecting HN-specific antibodies. For example, the presence of F- but absence of HN-specific antibodies indicates a vaccine response, while the presence of HN antibodies indicates exposure to wild viruses. HN-specific antibodies can be detected using traditional hemagglutination inhibition (HI) methods. As a traditional serological method, the protocol of the HI test is simple and presents no special requirements for the skills of operators. In addition, we have previously established an immunochromatographic strip to detect antibodies that recognize the HN and F protein of Newcastle disease virus. The strip can quickly distinguish F-vaccinated animals from natural-infected animals within 10 minutes. 

With the growing demand for animal protein, coupled with increasing concerns about animal welfare, microbial resistance to antibiotics, and food safety, the focus of poultry health has shifted from treatment to prevention. The plant F vaccine developed in this study is safer, and there is no need to worry about poultry infections caused by improper vaccine administration regimens. Moreover, the subunit vaccines would also achieve a DIVA strategy to allow the differentiation of infected birds from vaccinated birds. The use of 4.5 μg/dose provided immune protection for chickens in this study, showing that the low cost of the F vaccine can meet the needs of the market. In addition, rice as a bioreactor can be stored for long periods of time providing good control of production scale. In conclusion, our study provides evidence that transgenic rice can achieve high accumulation of recombinant protein with correct modifications and folding. Plant-produced F vaccine is safe, efficient, and inexpensive. The plant-derived F vaccine along with the immunochromatographic strips could be useful in the implementation of an NDV eradication program.

## 5. Conclusions

In this study, we provided a new transgenic rice-based ND subunit vaccine and a rapid differential diagnostic platform. Compared to traditional live vaccines, plant produced F vaccine is safer and has no adverse effects on chickens. Two doses of 4.5 μg fully protected chickens from a lethal ND challenge without any clinical symptoms. In addition, the rapid detection platform in this study can distinguish vaccine-immunized animals from naturally infected animals within ten minutes. Our study provides evidence that transgenic rice is a promising bioreactor. The plant-produced F vaccine along with the immunochromatographic strips could be useful for ND eradication.6. Patents

In this study, we have applied to the State Intellectual Property Office of the People’s Republic of China for a patent of “an immunochromatographic strip to detect antibodies targeting HN protein of NDV”. The publication Patent Number is CN 110018304 A.
