# Correction: Ma et al. A Plant-Produced Recombinant Fusion Protein-Based Newcastle Disease Subunit Vaccine and Rapid Differential Diagnosis Platform. *Vaccines* 2020*, 8,* 122

**DOI:** 10.3390/vaccines13050504

**Published:** 2025-05-09

**Authors:** Fanshu Ma, Erqin Zhang, Qingmei Li, Qianru Xu, Jiquan Ou, Heng Yin, Kunpeng Li, Li Wang, Xiangyue Zhao, Xiangxiang Niu, Xueyang Li, Shenli Zhang, Yanan Wang, Ruiguang Deng, Enmin Zhou, Gaiping Zhang

**Affiliations:** 1 Department of Preventive Veterinary Medicine, College of Veterinary Medicine, Northwest A&F University, Yangling 712100, China; marfanshu@nwafu.edu.cn (F.M.); xuqianru@nwafu.edu.cn (Q.X.); zhouem@nwsuaf.edu.cn (E.Z.); 2 Department of Preventive Veterinary Medicine, College of Animal Science and Veterinary Medicine, Henan Agricultural University, Zhengzhou 450002, China; zhangerqin76@163.com (E.Z.); nxx188@foxmail.com (X.N.); lxy4241@foxmail.com (X.L.); zhangshenli203814@foxmail.com (S.Z.); 3 Key Laboratory of Animal Immunology, Henan Academy of Agricultural Sciences, Zhengzhou 450002, China; liqingmeis@gmail.com (Q.L.); nongkewangli@163.com (L.W.); xiangyuezhao@foxmail.com (X.Z.); yanan18@mails.jlu.edu.cn (Y.W.); rgd999@163.com (R.D.); 4 Wuhan Healthgen Biotechnology Corp., Wuhan 430074, China; jqou@oryzogen.com (J.O.); yhen@oryzogen.com (H.Y.); lkp@oryzogen.com (K.L.)

The authors would like to make the following correction to this published paper [1].

In the original publication, in Section 2.3 “SDS-PAGE and Western Blotting”, the citation “Li et al., 2016” was inadvertently omitted from the reference list. This reference should be added to the reference list as No. 17.

17. Li, Q.; Wang, L.; Feng, L.; Zhang, Y.; Zhao, D.; Yang, Y.; Xing, G.; Cai, S.; Li, S.; Zhang, L.; et al. Production and Characterization of Neutralizing Monoclonal Antibodies against Newcastle Disease Virus. *J. Henan Agric. Sci.* **2017**, *46*, 127. 

As a result, the original references 17–22 have been reordered as 18–23.

The authors state that the scientific conclusions are unaffected. This correction was approved by the Academic Editor. The original publication has also been updated.
